# Preoperative knee laxity is not associated with subjective knee function or revision surgery after primary anterior cruciate ligament reconstruction: An analysis of 5425 patients

**DOI:** 10.1002/ksa.70058

**Published:** 2025-09-04

**Authors:** Riccardo Cristiani, Christoffer von Essen, Eric Hamrin Senorski, Camilo P. Helito, Kristian Samuelsson, Karl Eriksson, Magnus Forssblad, Anders Stålman

**Affiliations:** ^1^ Department of Molecular Medicine and Surgery, Section of Sports Medicine Karolinska Institutet Stockholm Sweden; ^2^ Stockholm Sports Trauma Research Center (SSTRC) FIFA Medical Centre of Excellence, Sophiahemmet Stockholm Sweden; ^3^ Department of Health and Rehabilitation, Institute of Neuroscience and Physiology, Sahlgrenska Academy University of Gothenburg Gothenburg Sweden; ^4^ Hospital Sírio Libanês São Paulo São Paulo Brazil; ^5^ Grupo de Joelho, Instituto de Ortopedia e Traumatologia, Hospital das Clínicas HCFMUSP Faculdade de Medicina da Universidade de São Paulo São Paulo Brazil; ^6^ Department of Orthopaedics, Institute of Clinical Sciences, The Sahlgrenska Academy University of Gothenburg Gothenburg Sweden; ^7^ Department of Orthopaedics, Stockholm South Hospital Karolinska Institutet Stockholm Sweden

**Keywords:** ACL, anterior cruciate ligament, knee laxity, KOOS, revision surgery, subjective knee function

## Abstract

**Purpose:**

To determine whether preoperative knee laxity, as measured by the KT‐1000 arthrometer, was associated with subjective knee function preoperatively and at 1, 2 and 5 years, or with revision anterior cruciate ligament (ACL) reconstruction (ACLR) within 5 years of the primary surgery.

**Methods:**

Patients who underwent primary ACLR using a hamstring tendon autograft at the Capio Artro Clinic, Stockholm, Sweden, between January 1, 2005, and December 31, 2018, and had no associated ligament injuries, were identified. The KT‐1000 arthrometer (134‐N) was used to assess knee laxity preoperatively. The Knee injury and Osteoarthritis Outcome Score (KOOS) was obtained preoperatively and at 1‐, 2‐ and 5‐year follow‐ups. Patients who underwent revision ACLR at any hospital or clinic nationwide within 5 years of their primary surgery were identified via the Swedish Knee Ligament Registry. KOOS subscale scores between groups were compared using analysis of covariance (ANCOVA), while differences in revision ACLR rates were evaluated using Cox regression analysis.

**Results:**

A total of 5425 patients (54.0% male) with available preoperative KT‐1000 arthrometer measurements were included: side‐to‐side (STS) ≤ 2 mm, 1833 (33.8%); STS 3–5 mm, 2387 (44.0%); STS > 5 mm, 1205 (22.2%). The only significant differences in subjective knee function among the groups were observed in the preoperative KOOS Symptoms (STS ≤ 2 mm: 75.0 ± 17.4; STS 3–5 mm: 75.1 ± 17.5; STS > 5 mm: 76.4 ± 17.1; *P* = 0.03) and Pain (STS ≤ 2 mm: 78.9 ± 15.9; STS 3–5 mm: 79.4 ± 15.9; STS > 5 mm: 80.4 ± 15.2; *p* = 0.02) subscale. No additional significant differences were observed between the groups in any of the KOOS subscales at the preoperative assessment or at the 1‐, 2‐ or 5‐year postoperative follow‐ups. At 5 years postoperatively, the revision ACLR rates were 4.8% (89/1833) in the STS ≤ 2 mm group, 4.9% (118/2387) in the STS 3–5 mm group and 6.0% (73/1205) in the STS > 5 mm group. The hazard of revision ACLR within 5 years did not differ significantly from the reference group (STS ≤ 2 mm) for either the STS 3–5 mm group (hazard ratio [HR], 0.96; 95% confidence interval [CI], 0.69–1.35; *p* = n.s.) or the STS > 5 mm group (HR, 1.19; 95% CI, 0.89–1.60; *P* = n.s.). The incidence of medial meniscus injury increased progressively across the laxity groups from 20.7% in the STS ≤ 2 mm group, to 25.0% in the STS 3‐5 mm group and 31.9% in the STS > 5 mm group.

**Conclusions:**

The degree of preoperative knee laxity, as measured by the KT‐1000 arthrometer, was not associated with postoperative subjective knee function or revision ACLR within 5 years of primary surgery. Medial meniscus injuries were associated with greater preoperative knee laxity. The findings of this study suggest that preoperative arthrometric knee laxity should not be considered as a prognostic factor for ACLR outcomes.

**Level of Evidence:**

Level III.

**Abbreviations**
ACL: anterior cruciate ligament
ACLR: anterior cruciate ligament reconstruction
ANCOVA: analysis of covariance
ANOVA: analysis of variance
CI: confidence interval
HR: hazard ratio
HT: Hamstring Tendon
IKDC: International Knee Documentation Committee
KOOS: Knee injury and Osteoarthritis Outcome Score
QOL: quality of life
SKLR: Swedish Knee Ligament Registry
SLH: Single Leg Hop
STS: side‐to‐side

## INTRODUCTION

The anterior cruciate ligament (ACL) serves as the main stabilizer against anterior displacement and internal rotation of the tibia [2, 19]. Among patients with ACL injuries, the degree of knee laxity can vary widely. Manual assessment remains the most commonly used method to evaluate laxity, and previous studies have reported that greater preoperative anterior knee laxity—defined as a Lachman test with more than 10 mm of side‐to‐side (STS) difference—was associated with a higher risk of revision surgery within 2 [23] and 6 years [24] after ACL reconstruction (ACLR). However, it is well established that manual laxity assessments are highly examiner‐dependent [31], potentially compromising their reliability. The KT‐1000 arthrometer (MEDmetric) is the most widely used device for assessing anterior knee laxity [4, 20]. It has been shown to be highly reliable [12, 18, 21, 38] and is considered superior to manual clinical examination, such as the Lachman test, as it offers an objective and quantitative measurement of anterior tibial translation [11, 37]. The International Knee Documentation Committee (IKDC) form [16] classifies knee laxity based on STS differences: ≤2 mm (normal), 3–5 mm (nearly normal) and >5 mm (abnormal). In a recent large cohort study [5], higher degrees of postoperative knee laxity (STS 3–5 mm and STS > 5 mm) assessed 6 months following primary ACLR were associated with an increased risk of revision ACLR within 5 years, although no correlation was found with subjective knee function. However, the association between preoperative arthrometric knee laxity and subjective knee function and the risk of revision surgery following primary ACLR remains poorly investigated. Gaining this knowledge could be highly valuable for patient counselling regarding prognosis and may also help guide treatment decisions.

The aim of this study was to evaluate whether preoperative arthrometric knee laxity was associated with subjective knee function preoperatively and at 1, 2, and 5 years following primary ACLR, as well as with the hazard of revision surgery within a 5‐year follow‐up period. The hypothesis was that increased preoperative knee laxity (STS 3–5 mm and STS > 5 mm) would be associated with poorer subjective knee function and a higher likelihood of revision ACLR.

## MATERIALS AND METHODS

### Patients

Ethical approval for this study was obtained from the regional ethics committee, Karolinska Institutet (Dnr 2016/1613‐31/32). The Strengthening of the Reporting of Observational Studies in Epidemiology statement was used as guidance to report this study to increase scientific quality [36].

Patients who underwent primary ACLR using a hamstring tendon (HT) autograft at the Capio Artro Clinic, Stockholm, Sweden, between January 1, 2005, and December 31, 2018, and had no associated ligament injuries, were retrospectively identified. Patients with a previous contralateral ACL injury or reconstruction, as well as those lacking preoperative arthrometric measurements, were excluded.

### Arthrometric knee laxity evaluation

Knee laxity was evaluated preoperatively at our outpatient clinic using the KT‐1000 arthrometer (MEDmetric), applying a standard anterior tibial load of 30 pounds (134‐N) with the knee at a 20‐degree flexion angle. Sports medicine physical therapists conducted all laxity assessments. Each knee was measured at least three times, and the median value was recorded. The difference in STS laxity (injured knee—healthy knee) was recorded. Knee laxity based on STS values was categorized into three groups (≤2 mm, 3–5 mm and >5 mm) as per the IKDC examination form [16].

### Surgical technique and rehabilitation

A single‐bundle technique was employed for all procedures. The semitendinosus tendon was primarily harvested and prepared as a tripled or quadrupled graft. If the tendon was insufficient in length or diameter (<8 mm), the gracilis tendon was additionally harvested and combined with the semitendinosus graft. An anteromedial portal technique was utilized to drill the femoral tunnel. The graft was routinely fixed with an Endobutton fixation device (Smith & Nephew) on the femoral side and with Ethibond No. 2 sutures (Ethicon) tied over an AO bicortical screw with a washer on the tibial side. No lateral extra‐articular procedures (lateral extra‐articular tenodesis or anterolateral ligament reconstruction) were performed. For meniscal tears in the posterior horn and body, an all‐inside arthroscopic technique using Fast‐Fix suture anchor devices (Smith & Nephew) was employed. Tears in the anterior portion of the meniscus were repaired using an outside‐in technique with PDS 0 sutures (Ethicon). Patients followed a supervised, standardized postoperative rehabilitation protocol. Full weight‐bearing and range of motion were permitted for cases involving isolated ACLR or ACLR with meniscal resection. For those undergoing ACLR with concomitant meniscal repair, a hinged knee brace was worn for 6 weeks. During the first 2 weeks, the brace allowed 0°–30° of flexion, 0°–60° for Weeks 3 and 4, and 0°–90° for Weeks 5 and 6 postoperatively. Starting at Week 7, the brace was discontinued, and a full range of motion was encouraged. Quadriceps strengthening was restricted to closed kinetic chain exercises for the first 3 months. Patients were cleared to return to sports as early as 6 months after ACLR, provided they met the isokinetic knee muscle strength and single‐leg hop (SLH) test criteria (limb symmetry index ≥ 90%) [6, 7, 34].

### Isokinetic strength test and SLH test performance

Muscle strength and the SLH test were evaluated 6 months after surgery. Isokinetic concentric extension and flexion strength were measured bilaterally using the Biodex System 3 (Biodex Medical Systems) at an angular speed of 90° per second. The assessments were conducted within a range of motion from 90° to 10°. Prior to testing, patients completed a low‐resistance warm‐up on a stationary cycling ergometer for approximately 10 min. Verbal instructions were provided, and participants were given two to three practice attempts to familiarize themselves with the test. Each leg performed five maximal repetitions for both extension and flexion, and the peak torque values—the highest force generated—were recorded [7].

The SLH test was also conducted [27]. Participants were instructed to balance on one leg, jump forward as far as possible, and land on the same leg. A stable landing was required for the test to be considered valid. If the opposite leg touched down prematurely, if there were additional hops upon landing, or if balance was lost, the attempt was repeated. Verbal instructions were provided before testing, along with several practice trials. The test was first performed on the uninjured leg, followed by the injured leg. Each leg underwent three attempts, and the longest successful hop was recorded [7].

### Subjective knee function

Subjective knee function was assessed using the Knee injury and Osteoarthritis Outcome Score (KOOS) [29, 30] prior to surgery and 1, 2 and 5 years after primary ACLR. The KOOS includes five subscales: Symptoms, Pain, Activities of Daily Living, Sport and Recreation, and knee‐related Quality of Life (QOL). Each subscale is scored individually, ranging from 0 to 100, where 0 indicates the poorest outcome and 100 reflects the best possible result.

### Revision ACLR

Patients who underwent revision ACLR at any hospital or clinic in Sweden within 5 years of their primary surgery were identified through the Swedish Knee Ligament Registry (SKLR) [33], using their unique Swedish personal identity number [22].

### Data sources

Data retrieved from our clinic database included preoperative KT‐1000 arthrometric measurements, patient age at the time of primary ACLR, time interval between injury and primary surgery, sex, the presence of a cartilage or meniscal injury, and information about meniscal procedures, categorized as either repair or resection of the medial or lateral meniscus. Additional variables retrieved from our database included preinjury Tegner activity level, graft diameter, isokinetic strength for knee extension and flexion at 6 months and SLH test performance. The KOOS subscale scores were extracted from the SKLR.

### Statistical analysis

Statistical analyses were performed using the Statistical Product and Service Solutions software (SPSS, Version 25.0; IBM Corp). Descriptive statistics, including means with standard deviations and frequencies, were used to summarize the data. The normality of variable distributions was assessed, and comparisons between continuous variables were conducted using analysis of variance (ANOVA), while Pearson's *χ* ^2^ test was applied for categorical variables. KOOS subscale scores at the preoperative, 1‐, 2‐ and 5‐year postoperative time points were compared among the laxity groups using analysis of covariance (ANCOVA). Medial meniscus injury was included as a covariate for preoperative KOOS values, while medial meniscus resection and medial meniscus repair were included as covariates in the postoperative analyses. These covariates were included in the analyses due to observed differences among the groups and because prior studies have indicated that these variables may influence KOOS subscale scores both preoperatively and postoperatively following ACLR [6, 8, 26, 32]. A Cox regression analysis was performed to compare the hazard of revision ACLR within 5 years of the primary surgery among the different laxity groups. The STS ≤ 2 mm group served as the reference group in the analysis. Hazard ratios (HRs) were adjusted for time from injury to primary ACLR, medial meniscus resection and medial meniscus repair. These confounding variables were included in the model due to observed differences among the laxity groups and because prior research has suggested that they may influence the risk of revision ACLR [3, 28]. The level of significance in the analyses was 5% (two‐tailed).

## RESULTS

A total of 7042 patients were screened for inclusion. Following the exclusion of patients with a prior ACL injury or reconstruction on the contralateral knee (*n* = 241) and those lacking preoperative KT‐1000 arthrometer data (*n* = 1376), 5425 patients met the eligibility criteria. These patients were then categorized into three groups based on their preoperative KT‐1000 arthrometer measurements, in line with the IKDC knee examination form: ≤2 mm, 3–5 mm and >5 mm STS difference. A complete overview of patient characteristics is provided in Table 1.

**Table 1:** Patient characteristics.

	STS ≤ 2 mm	STS 3‐5 mm	STS > 5 mm	*p* value
Patients, *n*	1833 (33.8)	2387 (44.0)	1205 (22.2)	
STS difference, mm	0.8 ± 1.6	3.9 ± 0.8	7.4 ± 1.5	<0.001
Age at surgery, mean ± SD, year	28.5 ± 10.7	27.8 ± 10.6	28.3 ± 10.9	n.s.
Time from injury to surgery, mean ± SD, mo	18.6 ± 40.4	18.9 ± 38.0	25.1 ± 44.4	<0.001
Patients with available data	1801	2361	1195	
Sex				n.s.
Male	1019 (55.6)	1255 (52.6)	657 (54.5)	
Female	814 (44.4)	1132 (47.4)	548 (45.5)	
Cartilage injury	317 (17.3)	404 (16.9)	225 (18.7)	n.s.
MM injury	379 (20.7)	597 (25.0)	385 (31.9)	<0.001
LM injury	424 (23.1)	554 (23.2)	309 (25.6)	n.s.
MM resection	220 (12.0)	370 (15.5)	246 (20.4)	<0.001
MM repair	99 (5.4)	169 (7.1)	105 (8.7)	0.002
LM resection	293 (16.0)	378 (15.8)	209 (17.3)	n.s.
LM repair	64 (3.5)	104 (4.3)	63 (5.2)	n.s.
Preinjury Tegner score, mean ± SD	7.2 ± 1.7	7.2 ± 1.7	7.1 ± 1.7	n.s.
Patients with available data	1749	2302	1159	
Graft diameter, mm	8.4 ± 0.8	8.4 ± 0.8	8.3 ± 0.7	n.s.
Patients with available data	1381	1841	979	
Postoperative (6 months) measurements				
Isokinetic extension strength, LSI, mean ± SD	86.5 ± 16.5	84.8 ± 14.9	84.5 ± 15.7	n.s.
Patients with available data	1568	2078	1037	
Isokinetic flexion strength, LSI, mean ± SD	90.9 ± 16.8	89.2 ± 14.8	88.4 ± 28.2	n.s.
Patients with available data	1567	2074	1036	
SLH test performance, LSI, mean ± SD	94.0 ± 12.5	93.0 ± 12.8	91.7 ± 12.7	n.s.
Patients with available data	1334	1805	874	

*Note*: Data are reported as number (percentage) unless otherwise indicated.

Abbreviations: LM, lateral meniscus; LSI, limb symmetry index; MM, medial meniscus; n.s., non significant; SD, standard deviation; SLH, single leg hop; STS, side to side.

### Subjective knee function

A total of 5418 patients (99.9%), 4158 patients (76.6%), 2967 patients (54.7%) and 2474 (45.6%) patients had available preoperative, 1‐year postoperative, 2‐year postoperative and 5‐year postoperative KOOS subscale scores, respectively.

The only significant differences in subjective knee function among the groups were observed in the preoperative KOOS Symptoms (STS ≤ 2 mm: 75.0 ± 17.4; STS 3–5 mm: 75.1 ± 17.5; STS > 5 mm: 76.4 ± 17.1; *P* = 0.03) and Pain (STS ≤ 2 mm: 78.9 ± 15.9; STS 3–5 mm: 79.4 ± 15.9; STS > 5 mm: 80.4 ± 15.2; *P* = 0.02) subscale. No other significant differences were observed between the groups in any of the KOOS subscales at the preoperative assessment or at the 1‐, 2‐ or 5‐year postoperative follow‐ups (Table 2).

**Table 2:** Preoperative and postoperative KOOS comparison.

	STS ≤ 2 mm	STS 3‐5 mm	STS > 5 mm	*p* value
*Symptoms*				
Preoperative	75.0 ± 17.4 (1831)	75.1 ± 17.5 (2383)	76.4 ± 17.1 (1204)	0.03
1‐year	79.9 ± 16.8 (1381)	80.7 ± 15.9 (1830)	81.7 ± 15.9 (947)	n.s.
2‐year	81.9 ± 16.4 (988)	82.3 ± 15.8 (1288)	83.1 ± 15.4 (691)	n.s.
5‐year	84.7 ± 15.7 (852)	83.1 ± 16.9 (1065)	84.1 ± 15.7 (557)	n.s.
*Pain*				
Preoperative	78.9 ± 15.9 (1831)	79.4 ± 15.9 (2383)	80.4 ± 15.2 (1204)	0.02
1‐year	87.4 ± 13.7 (1381)	88.1 ± 12.3 (1829)	88.6 ± 12.0 (947)	n.s.
2‐year	88.0 ± 13.9 (988)	88.3 ± 13.4 (1288)	88.6 ± 13.1 (691)	n.s.
5‐year	89.5 ± 13.4 (852)	88.3 ± 14.1 (1065)	88.8 ± 14.1 (557)	n.s.
*ADL*				
Preoperative	87.7 ± 14.1 (1831)	87.6 ± 14.4 (2383)	88.1 ± 14.3 (1204)	n.s.
1‐year	93.9 ± 10.9 (1381)	94.3 ± 9.6 (1830)	94.7 ± 9.1 (947)	n.s.
2‐year	93.5 ± 10.4 (988)	94.1 ± 10.6 (1288)	94.1 ± 11.3 (691)	n.s.
5‐year	94.5 ± 10.4 (852)	94.0 ± 11.1 (1065)	93.9 ± 10.9 (557)	n.s.
*Sport/Recreation*				
Preoperative	48.3 ± 28.8 (1830)	48.2 ± 28.1 (2383)	48.4 ± 27.7 (1204)	n.s.
1‐year	70.8 ± 25.0 (1381)	71.5 ± 24.1 (1830)	71.2 ± 24.5 (947)	n.s.
2‐year	72.0 ± 26.4 (988)	73.3 ± 24.0 (1288)	73.4 ± 24.5 (691)	n.s.
5‐year	74.3 ± 26.3 (851)	72.7 ± 26.3 (1064)	73.2 ± 26.0 (557)	n.s.
*QOL*				
Preoperative	38.1 ± 22.7 (1830)	37.3 ± 22.0 (2383)	36.8 ± 22.2 (1204)	n.s.
1‐year	62.3 ± 23.5 (1381)	62.2 ± 22.7 (1830)	61.9 ± 23.4 (947)	n.s.
2‐year	66.5 ± 24.1 (988)	66.3 ± 22.8 (1288)	66.9 ± 23.4 (691)	n.s.
5‐year	70.0 ± 24.3 (852)	68.0 ± 24.8 (1065)	68.5 ± 24.7 (555)	n.s.

*Note*: Data are reported as mean ± SD (*n*). Covariates applied to the model (analysis of covariance) are medial meniscus injury (preoperative KOOS) and medial meniscus resection and medial meniscus repair (postoperative KOOS).

Abbreviations: ADL, activities of daily living; KOOS, Knee injury and Osteoarthritis Outcome Score; QOL, quality of life; STS, side to side.

### Revision ACLR

The 5‐year revision ACLR rates following primary surgery were 4.8% (89 out of 1833) for patients with an STS of ≤2 mm, 4.9% (118 out of 2387) for those with an STS of 3–5 mm, and 6.0% (73 out of 1205) for patients with an STS greater than 5 mm. The hazard of undergoing revision ACLR within 5 years was not significantly increased in the 3–5 mm or >5 mm STS groups when compared with the ≤ 2 mm group, as shown in Table 3.

**Table 3:** Relationships between preoperative STS laxity and revision ACLR within 5 years from primary surgery.

	HR (95% CI)	*p* value
STS ≤ 2 mm	1 (reference group)	
STS 3–5 mm	0.96 (0.69–1.35)	n.s.
STS > 5 mm	1.19 (0.89–1.60)	n.s.

*Note*: Covariates applied to the model (Cox regression analysis) are time from injury to surgery, medial meniscus resection, and medial meniscus repair.

Abbreviations: ACLR, anterior cruciate ligament reconstruction; CI, confidence interval; HR, hazard ratio; STS, side to side.

### Secondary findings

The incidence of medial meniscus injury increased progressively across the laxity groups from 20.7% in the STS ≤ 2 mm group, to 25.0% in the STS 3–5 mm group and 31.9% in the STS > 5 mm group.

## DISCUSSION

The most important finding of this study was that the degree of preoperative knee laxity, as measured by the KT‐1000 arthrometer, was not associated with postoperative subjective knee function or the hazard of revision ACLR within 5 years of primary ACLR. No significant differences were observed between the preoperative laxity groups in any of the KOOS subscales at the 1‐, 2‐ or 5‐year postoperative follow‐ups. The only significant differences were observed in the preoperative KOOS Symptoms (STS ≤ 2 mm: 75.0 ± 17.4; STS 3–5 mm: 75.1 ± 17.5; STS > 5 mm: 76.4 ± 17.1; *p* = 0.03) and Pain (STS ≤ 2 mm: 78.9 ± 15.9; STS 3–5 mm: 79.4 ± 15.9; STS > 5 mm: 80.4 ± 15.2; *p* = 0.02) subscale. However, these differences were minimal and well below the threshold commonly considered clinically relevant (<8–10 points) [29]. The 5‐year revision ACLR rates following primary surgery were 4.8% for patients with an STS of ≤2 mm, 4.9% for those with an STS of 3 to 5 mm and 6.0% for patients with an STS greater than 5 mm. The hazard of undergoing revision ACLR within 5 years was not significantly increased in the 3–5 mm or >5 mm STS groups when compared with the ≤2 mm group.

Only a limited number of studies have explored the association between instrumented preoperative anterior knee laxity and subjective knee function. Eitzen et al. [13] found no correlation between anterior laxity—measured with the KT‐2000 arthrometer—and the Cincinnati knee score 2 years after ACLR in a cohort of 60 patients. Similarly, Heijne et al. [17] reported no significant association between preoperative KT‐1000 values and the KOOS Sport/Recreation or QOL subscales, nor the Tegner activity scale, 1 year postoperatively in a cohort of 64 patients. While these earlier studies were limited by small sample sizes and may have been underpowered to detect potential associations (raising the possibility of type II error), our study—representing, to our knowledge, the largest cohort to date to investigate this question—found no evidence of a relationship between the degree of preoperative KT‐1000 STS difference and postoperative KOOS subscale scores.

Interestingly, a high grade of preoperative knee laxity (STS 3–5 and > 5 mm) was not associated with an increased hazard of revision ACLR within 5 years of the primary surgery. Previous studies investigating the relationship between preoperative manual anterior knee laxity and the risk of revision ACLR have yielded conflicting results. Magnussen et al. [23] reported that high‐grade preoperative anterior knee laxity, defined as Lachman grade D, was associated with higher odds of revision ACLR within 2 years. A subsequent follow‐up of the same patient cohort [24] demonstrated that patients with preoperative Lachman grade D had increased odds of revision ACLR 6 years after the primary surgery. However, it is important to note that these studies [23, 24] included a variety of graft types—bone–patellar tendon–bone autograft (44.7%), HT autograft (37.9%), quadriceps tendon autograft (0.1%) and allograft (17.4%)—which may have biased the results. Comparisons between studies are challenging due to variations in surgical techniques, graft selection, rehabilitation protocols and—most importantly—the methods used to assess laxity (manual testing vs. KT‐1000 arthrometer). The present study, which analyzed a large patient cohort, employed a single graft type (HT autograft) and the KT‐1000 arthrometer—an objective tool for measuring anterior knee laxity—found no association between the degree of preoperative knee laxity and the hazard of revision ACLR within 5 years of the primary surgery. A recent large cohort study [5] found that high‐grade postoperative knee laxity (STS 3–5 mm and STS > 5 mm), measured 6 months after primary ACLR, was associated with an increased hazard of revision ACLR within 5 years. Based on these findings, it can be concluded that achieving postoperative knee laxity levels as close as possible to those of the native knee (STS ≤ 2 mm) is more important in reducing the hazard of revision ACLR than the degree of knee laxity prior to primary ACLR.

Finally, it is interesting to note that the incidence of medial meniscus injury increased progressively across the laxity groups. The highest incidence of medial meniscus injury (31.9%) was observed in the group with the greatest preoperative laxity (STS > 5 mm). This finding supports the critical function of the medial meniscus in limiting anterior translation of the tibia [9, 14, 35].

The large sample size (5425 patients) was a major strength of this study, allowing for robust analysis of covariance and Cox regression analyses to compare subjective knee function and the hazard of revision ACLR across groups and ensuring high generalizability of the results. All patients underwent surgery at the same institution and followed a standardized rehabilitation protocol, minimizing the influence of these factors as potential confounders on the outcomes assessed. Another strength was the exclusion of patients with concomitant knee ligament injuries and the inclusion of only HT ACLRs, thereby minimizing the influence of associated injuries and graft type on knee laxity, subjective outcomes and the risk of revision ACLR [1, 4, 6, 10, 15, 25]. Knee laxity was evaluated using the KT‐1000 arthrometer, which provides a quantitative measure of anterior tibial translation and is regarded as more accurate than manual assessment [11, 37]. To the best of our knowledge, this is the first study to explore the impact of preoperative arthrometric laxity measurements on subjective knee function and the hazard of revision surgery following primary ACLR in a large patient cohort. Finally, the SKLR allowed us to track patients who underwent revision ACLR at any healthcare facility nationwide.

This study has several limitations. First, a commonly cited concern with KT‐1000 arthrometer assessments is the potential for interobserver variability, as results can be influenced by the examiner's proficiency. However, several factors helped to minimize this potential issue: (1) the assessments were performed by experienced physical therapists who routinely used the KT‐1000 arthrometer; (2) a standardized anterior tibial load of 134 N was applied, with each knee measured at least three times; and (3) the large sample size further mitigated the impact of any potential variability. Second, the effect of rotatory knee laxity on the study outcomes was not assessed as the pivot‐shift test is not registered in a standardized way in our registry. However, it is widely recognized that assessment of rotatory knee laxity can vary considerably between examiners due to its reliance on the examiner's skill, making it less reliable. Third, the KOOS follow‐up rates at 2 years (54.7%) and 5 years (45.6%) were suboptimal. Loss to follow‐up for patient‐reported outcome measures is a well‐documented issue in ACL registries, and these rates are consistent with those reported in previous large cohort studies [6, 8, 32]. Nonetheless, 2‐ and 5‐year KOOS subscale scores were available for 2967 and 2474 patients, respectively, providing a very high statistical power. Fourth, the outcome assessed in this study was revision ACLR rather than graft rupture, as this information is not available in the SKLR. Therefore, potential differences in graft rupture rates between the laxity groups could not be evaluated. Another limitation was that we lacked data on return to sport and postsurgical activity levels. Potential differences between groups in these factors may have influenced the hazard of revision ACLR. However, since no differences were observed in preinjury Tegner scores between groups, any variation in return to sport or activity levels would likely have affected all groups similarly, minimizing the risk of systematic bias. Finally, postoperative arthrometric knee laxity data were not included. However, a recent large cohort study has already explored the relationship between postoperative KT‐1000 measurements, subjective knee function and the hazard of revision ACLR [5]. The primary objective of the present study was to specifically investigate the association between preoperative arthrometric knee laxity and these outcomes.

## CONCLUSION

The degree of preoperative knee laxity, as measured by the KT‐1000 arthrometer, was not associated with postoperative subjective knee function or revision ACLR within 5 years of primary surgery. Medial meniscus injuries were associated with greater preoperative knee laxity. The findings of this study suggest that preoperative arthrometric knee laxity should not be considered as a prognostic factor for ACLR outcomes.

## AUTHOR CONTRIBUTIONS

**Riccardo Cristiani:** Conceptualization; data curation; methodology; writing. **Christoffer von Essen, Eric Hamrin Senorski, Camilo P. Helito, Kristian Samuelsson, Karl Eriksson, and Magnus Forssblad:** Methodology and critical review of the manuscript. **Anders Stålman:** Conceptualization; methodology and critical review of the manuscript. All authors have given final approval of the version to be published.

## CONFLICT OF INTEREST STATEMENT

The authors declare no conflicts of interest.

## ETHICS STATEMENT

Ethical approval for this study was obtained from the regional ethics committee, Karolinska Institutet, Diarenumber 2016/1613‐31/32. No consent to participate is requested for registry studies.

## Data Availability

The data that support the findings of this study are available from the corresponding author [R.C.], upon reasonable request.
